# Training Working Memory of Children with and without Dyslexia

**DOI:** 10.3390/children6030047

**Published:** 2019-03-20

**Authors:** Claudia Maehler, Christina Joerns, Kirsten Schuchardt

**Affiliations:** Department of Educational Psychology, Institute of Psychology, Universitätsplatz 1, 31141 Hildesheim, Germany; joerns@uni-hildesheim.de (C.J.); schuchar@uni-hildesheim.de (K.S.)

**Keywords:** working memory training, dyslexia, elementary school-age

## Abstract

For the future school performance of a child in the fields of literacy and numeracy, the operational efficiency of working memory is a central predictor. Children affected by dyslexia exhibit specific deficits in the functions of working memory. A software application for elementary school-age children has been specifically developed for this study, attempting to improve the working memory’s operational efficiency. Based on Baddeley’s model of working memory (1986), the phonological loop, the visuo-spatial sketchpad, and the central executive were trained in 18 sessions over a period of six weeks. The group of test subjects undergoing this training was composed of third-graders, of which 43 were and 27 were not affected by dyslexia. The untrained control group was made up of 41 third-graders with dyslexia and 28 without dyslexia. While the short-term effects of the program could not be proven, the present analyses focus on long-term effects. The results obtained from a pre-test/follow-up design reveal that no long-term increases in performance regarding phonological and central executive working memory could be confirmed. Only the visuo-spatial Corsi block span exhibited a training effect over a period of three months. Additionally, training did not show any long-term effect of performance improvement, not even for a subgroup of children with dyslexia and an especially low working memory performance. Thus, even after this study, the question whether working memory can be trained or not remains partly unanswered but leaves us predominantly pessimistic.

## 1. Introduction

In search of aid and support for children with difficulties in the acquisition of written language, a multitude of approaches has been put forth and tested for many years.

Until now, most effective programs directly used reading and writing as forms of functional exercise training [1]. Aside from that, attempting to positively impact the suspected causes of written language acquisition impairment may be worth its while. In this vein, the present study’s goal is to improve the operational efficiency of working memory as a possible etiological factor.

### 1.1. Characteristics of Dyslexia

Difficulties in reading and writing (dyslexia) are characterized by typical patterns. Reading problems become evident from the very start of the child’s school career, through difficulties in reciting the alphabet or through delayed or incorrect character recognition. The child’s reading comprehension, the ability to recognize words and to read them out, is impaired. Reading errors occur, such as omissions, replacements, inversion, or additions of words or word parts, and also the mixing-up of parts of words within a sentence. A significantly reduced reading speed can be detected. Overall, the reading process requires great amounts of effort and concentration. Orthographical problems commonly arising in the context of German written language are associated with switches in the order of characters, character omission or character insertion within a word, orthographic errors (e.g., disregard of capitalization rules, doubling of characters or lengthening), and perception errors (e.g., confusing phonemes such as d/t, b/p, or g/k).

The characteristics for these orthographical problems are not so much typical errors, but rather a large quantity of errors along with an error inconstancy [2].

Studies with a longitudinal design exhibit a higher temporal stability of the defined dyslexic impairments, frequently up into adulthood [3]. While affected children improve overall in their literary language performances, they nevertheless lag behind compared to their unimpaired fellow students. This relatively high stability of literary language acquisition impairment is evidence to suggest a relatively stable cause of the dysfunction.

### 1.2. Information Processing Problems

Several causes for dyslexia are under discussion, such as genetic and brain-related parameters, adverse environmental conditions and personality traits, as well as cognitive factors [1]. Since these different causes do not apply to the same degree for every individual child, it is not unlikely to produce the rather heterogenous presentation of dyslexia. So far, causal research has especially paid much attention to the search for cognitive deficits. Research efforts in this context increasingly focus on deficits in phonologic processing and memorization. It is no longer controversial that dyslexia constitutes a phonological information processing disorder [4,5,6]. Processing of phonological information describes the use of phonological structures of information in the interaction with spoken and written language [7]. This can be subdivided into three factors: phonological awareness, phonological recoding in the working memory and the recalling of phonological codes from long-term memory. A multitude of studies has been dealing with the difficulties children with dyslexia have regarding memory tasks. 

Baddeley’s model of working memory [8,9] has proven quite helpful as a theoretical basis for the identification of specific impairments within the respective pathologies, for instance dyslexia, dyscalculia, language impairment, attention-deficit hyperactivity disorder, or autism [4,10,11]. Baddeley [8,12] differentiates between three subsystems of working memory—a central executive, a phonological loop and a visuo-spatial sketchpad—each with its individual sphere of responsibility. The central executive assumes the task of controlling and monitoring cognitive processes, for example the coordination of resources while handling multiple chores, the flexible recall of long-term memory information, alternating between different strategies of memory, or controlling selective attention [13]. It is often measured by complex span tasks, such as remembering information and recalling it backwards. Hence, the central executive’s function is largely compatible with other theoretical models of a general working memory, being responsible for information processing, while equipped with a limited capacity [14,15]. The working memory’s central executive is assisted by two domain-specific subsystems. Processing of auditory information is accomplished through the phonological loop, which is, in turn, comprised of phonological store and a process of internal speaking (rehearsal process). The capacity of the phonological loop is usually measured via a serial reproduction of verbal information (e.g., word span or number span). Finally, the processing of static and dynamic spatial information is carried out by the visuo-spatial sketchpad, in charge of recalling visual material which is not processable through the phonological recoding routines the phonological loop performs. Tasks designed for measuring the visuo-spatial sketchpad will typically require recalling pictures devoid of content or spatial positions [16].

### 1.3. Working Memory Deficits 

An impairment of the phonological working memory in children suffering from dyslexia has been proven in several studies [11,17]. In this case, the entire system of the phonological loop seems to be affected, i.e., the deficits of children with dyslexia manifest themselves both in tasks regarding the phonological memory, as well as in tasks which put a stronger emphasis on the rehearsal component [11]. Beyond that, there is early evidence that subgroups of children with an impaired literary language acquisition might differ in deficits of their working memory. Apparently, memorizing words devoid of meaning (non-words) is particularly difficult for children with reading deficits, while children with spelling disorders display special limitations when it comes to longer phonetic sequences [18]. In addition, evidence can be found for deficits in the central executive’s functions [19,20,21,22,23,24]. Recent analyses also illustrate that deficits of the phonological loop can predominantly be found in children affected by spelling deficits, whereas children with reading difficulties exhibit functional issues of the central executive [17].

However, impairments of visuo-spatial working memory performance are seldomly reported for children with dyslexia [4,25,26,27,28].

Concluding from the research results depicted thus far, dyslexia is accompanied by working memory deficits. These deficits are localized particularly in the phonological loop and can be understood as a causal problem in the context of other phonological impairments (deficits of phonological awareness and of accessing the semantic lexicon).

### 1.4. Working Memory Training

The fact that not only children with dyslexia, but also children suffering from other learning impairments or attention deficit disorders are affected by working memory interferences gives rise to the question whether working memory can be subject to training. For a long time, the prevailing view has been that memory performance could only be enhanced through the accumulation of knowledge, through memory strategies and metacognitive control. Attempts to enhance the working memory’s capacity through mere repetitive practice for the automatization of rehearsal have not proven to be very successful [29].

Only recently, initial research efforts have been made in the form of target-oriented working memory training, focused on increasing memory performance and the examination of transfer effects. Meanwhile, research on working memory training is in full swing, since the acknowledgment of the working memory’s significance for children’s receptiveness and overall school performance has intensified the endeavors of specifically promoting this field of cognitive functions. However, instead of studies on dyslexic children, research on unaffected children and adults, as well as on individuals suffering from attention-deficit/hyperactivity disorder (ADHD) are the center of the research’s attention [30,31]. Recently published studies deal with children with learning disabilities (and working memory deficiencies), whose working memory performance has been successfully improved via adaptive training over the course of the study [32]. These surveys differ considerably regarding their respective objectives of interventions. Frequently, the emphasis lies on training methods aimed at the optimization of central executive control, instead of on the phonological loop or on the visuo-spatial sketchpad [33].

The current state of the art concerning the efficiency of such working memory training is characterized by its pronounced heterogeneity—it ranges from exceptionally optimistic prognoses to rather pessimistic estimations, with the latter predicting the utter ineffectiveness of the previously outlined programs [31,34]. Commercially distributed programs such as CogMed [30] and Jungle Memory [35] are the subject of particular praise and recommendation due to their alleged usefulness, even though no convincing long-term proof of efficiency or transfer effects have been presented so far. Two extensive and thorough meta-analyses [31,34], dealing with a variety of current training studies, reach a rather critical verdict: While short-term performance improvements in verbal and visual working memory are feasible, these increases in efficiency cannot be maintained on a long-term basis, they will be lost with certainty for the verbal realm and overwhelmingly for the visual part. No reliable transfer effects for verbal capacity, written language, or mathematics could be detected. These results, however, have for the better part been yielded from studies with unaffected children. Children with lower working memory capacities were able to improve their performances when exposed to adaptive training patterns, designed to encourage them to constantly train at their individual limits—but also for this setting, no significant transfer could be determined [32].

Shipstead, Hicks and Engle [36] have given a succinct account of the present state of research and its recent controversial condition: They suggest maintaining an open outcome-oriented research strategy in order to explore the conditions, potentialities, and limitations of working memory training. The fact that not every study published so far has been convincing does not, on the one hand, render a successful training of these cognitive capacities impossible, while on the other hand, one should be careful not to unreflectingly establish programs not yet evaluated for effectiveness. The present study evaluates the long-term effects of a training program which is design-wise strongly oriented towards the theoretical foundation of Baddeley’s model [8]. The study has been conducted with groups of elementary school-age children with and without dyslexia. The short-term effects immediately after the training sessions have already been reported [37]. The findings substantiate performance improvements for the group of typically developing third-graders in the subsystems visuo-spatial sketchpad and central executive, for the children with dyslexia only in the central executive. Unfortunately, and despite intensive training efforts, effects failed to materialize for the phonological loop, a part of the working memory crucial for children affected by dyslexia.

The analyses portrayed here examine the question whether the working memory’s functional efficiency can be improved lastingly for both dyslexic and typically developing children, i.e., whether training effects can be maintained over a longer period or might even occur after a certain period of time (sleeper effect). This first research question requires the comparison of trained and untrained children with and without dyslexia a few months after the training. A second research questions is concerned with the finding that children with lower working memory capacities seem to profit more from an adaptive training than those with better capacities [32]. It is a well-known phenomenon that differential training effects result in relation to the individual competencies before an intervention. And even if any training might not be beneficial for all participants, improvements in the most affected subgroup might point to a valuable contribution of the intervention. Subsequently, the present study investigates whether dyslexic children with notably poor working memory performance benefit from the training, i.e., show long-term effects with regard to their working memory performance.

## 2. Methods

### 2.1. Participants

A total of 139 third-graders participated in this present study, both from rural and urban areas. Recruiting took place in primary schools and also in our university counseling center for learning disabilities. The participants were assigned the following groups: Group “dyslexia” (subgroups: DYS-T = dyslexia trained or DYS-UT = dyslexia untrained), and one of two control groups (C-T = control group trained and C-UT = control group untrained). For this study, the inclusion criteria of dyslexia are a substandard reading and/or writing performance (T-value < 40) combined with at least an average intelligence (IQ ≥ 85). The children of both control groups exhibited at least average school and intelligence performance values (T > 40). Table 1 contains the sample parameters—sex and age, as well as intelligence scores and the results of the standardized reading and writing tests.

### 2.2. Design and Instruments

All children were examined within a pre-test, post-test, follow-up design (PPF) at three test intervals either in their schools or in our university counseling center. Assessment was done by well-trained student research assistants. The first measurement (pre-test) was taken at the beginning of the study in order to assess school performance, intelligence and working memory capacity. Subsequently, over a period of six weeks, three weekly training sessions of 45 minutes were conducted—either in small training groups in our counseling center or as a class in the primary schools. Post-testing was done after the end of training and has been reported elsewhere [37]. Three months later, follow-up measurements were taken, once more recording working memory performance to determine the therapy’s long-term efficacy. The collection of learning levels for pre-test measurements was either made within the classes of the participating schools or in smaller groups in our counseling center. For the general intelligence assessment, the Culture Fair Intelligence Test, Scale 1 (CFIT [38]) was carried out. School performances in the field of reading were collected employing the reading comprehension test for first- to six-graders (ELFE 1–6 [39]). The Weingartener spelling test (WRT 2+ [40]) was used to assess written language capacities.

In an individual test setting, the adaptive working memory test battery for children from ages five to twelve (AGTB 5–12 [41]) was applied to survey the working memory’s performance. Validity of this training battery, especially its relation to school achievement, has been examined in several studies, reported in the manual of the test [41]. Split-half reliability scores were reported here for every single subtest. Phonological loop: For the tasks *one-syllable word span* (r = 0.95), *three-syllable word span* (r = 0.92) and *digit span* (r = 0.96), audio recordings of increasing sequences of familiar monosyllabic words (e.g., Stern, Fisch, Ball (star, fish, ball)), respectively trisyllabic (e.g., Erdbeere, Briefkasten, Kneifzange (strawberry, mailbox, pincers)), respectively numerals (1–9) were played to and immediately verbally repeated by the child in the same order. Visuo-spatial sketchpad: For the *Corsi block* task (r = 0.99), the child was presented with nine unsystematically arranged white squares on a touchscreen display, in which, in short succession, small emoticons pop up. Following this presentation, the child was instructed to enter the squares into the touchscreen in the same order. For the task *Matrix* (r = 0.96), patterns of black and white sections of varying complexity were presented in a 4-by-4 matrix on screen, starting with two and gradually increasing to a maximum of eight black sections. Immediately after the presentation, the children were encouraged to reproduce the black sections on an empty screen. Central executive: In the task *object span* (r = 0.96), different objects (e.g., ball, candy, candle) appeared on a touchscreen display; the child was asked to judge the objects’ edibility and subsequently repeat the objects in the correct order. The task *digit span backwards* (r = 0.96) consisted of increasing sequences of acoustically presented numerals, which, in contrast to the forward span, were repeated in inverse order. The task *color span backwards* (r = 0.97) displayed a succession of monochrome circles on the computer; subsequently, they were to be touched on the screen in inverse order. In the subtest *counting span* (r = 0.97), consecutive arrangements of blue circles and squares appeared on the display. The child’s task was to count and memorize only the circles. The end of one cycle was followed by the reproduction of the number of circles counted in correct order. The task *Corsi block inverse* (r unknown because the task was not part of the original battery) was executed analogously to the *Corsi block* task (see above), with the difference that the child was—after having been shown the sequence—asked to enter the squares on the display in the inverted order.

Initially, to assess individual working memory performance, the mean of the two longest correctly reproduced sequences was determined for every subtest (raw score). Subsequently, in order to determine paramount scores for all three subsystems of working memory, a mean for each subsystem was derived from the raw scores of those subtests belonging to the respective subsystem.

### 2.3. Working Memory Training

An adaptive training program for the improvement of both capacity and operational efficiency of working memory has been developed. The training by the name of AGENT 8–1–0 (Arbeitsgedächtnistraining für Kinder von 8 bis 10 Jahren mit der ICD-Diagnose F 81.0 [Working memory training for children from ages eight to ten, respectively diagnosed with ICD 81.0]) was designed on the basis of the working memory test battery for children ages five to twelve (AGTB 5–12 [41]). Hence, the training closely follows Baddeley’s model [8], realizing it by means of a computer game in which children team up with private investigator Anton. AGENT 8–1–0 is comprised of 18 training sessions (divided into segments of three 45-min sessions per week over a course of six weeks). Training took place in the classes or in smaller groups in our counseling center and was administered by student research assistants. Every child was sitting in front of her own monitor, wearing earphones and working individually on the tasks. This is essential, because the program follows an adaptive algorithm related to the individual level of working memory capacity. Student research assistants accompanied the beginning for every child, but subsequently all instructions were given by the computer so that every child could proceed at her own pace. In each of the 18 training sessions, five working memory tasks out of a total of ten were assigned. These tasks were: two games aimed at improving the phonological loop’s capacity, one game for the improvement of the visuo-spatial sketchpad and two games for the stimulation of the central executive—of the latter, one containing phonologic and one containing visuo-spatial information. At the beginning, each individual game gave 15 trials of the same make-up, later, the number rose to 20.

The following paragraphs briefly describe the games.

*What Has Been Stolen Where?* (*Phonological loop*). Victims of a series of burglaries report their missing possessions to Anton. After listening to an audio recording listing a maximum of nine items (loot), the child is asked to reproduce the items’ correct order using the touchscreen. The first nine training units feature monosyllabic items, and the last three weeks include trisyllabic ones, which require a higher phonological loop capacity. Along with increasing task difficulty, the number of items increases.
*Come Again?* (*Phonological loop*). The child is to judge whether two audio recordings of pseudo-words (words of a secret language) feature the same word. The replayed words are either identical or show a slight difference in one of their phonemes. With an increased level of difficulty, the number of syllables increases (up to a maximum of seven).
*Open the Vault*. (*Phonological loop*). The child is visually presented with a code of up to nine different colors. If the combination is reproduced in the correct order, using a control panel, the vault opens. Each color appears only once. With higher levels of difficulty, the color sequence increases in length.
*Jewelry Store Mayhem.* (*Visual-spatial sketchpad*). The child sees a jewelry box with 20 drawers, and the box contains up to nine pieces of jewelry. Then, after the jewelry is stolen and restituted, the child has to organize the jewelry in its former order by clicking onto the right drawers. The higher the level of difficulty, the more pieces of jewelry have to be sorted.
*To Catch a Thief*. (*Visual-spatial sketchpad*). Twelve houses are arranged on a 4-by-4 grid. A thief appears in one house after the other, in a maximum of nine houses, indicating his escape route. Afterwards, the child is to reconstruct the getaway by clicking on the houses in the correct order. With a higher level of difficulty, the number of houses increases*. Mission accomplished.* As in the game
*What Has Been Stolen Where? (Central executive: phonological).* (see above) The child listens to a maximum of nine monosyllabic audio recordings, which have to be entered into the touchscreen in reverse order.
*A big haul. (Central executive: phonological).* In this game, a succession of up to nine audio recordings of disyllabic items is presented, which then have to be repeated in the right order. This time, the child is to additionally subsume each item under a category (school materials, food, clothing), which complicates the process of memorization.
*Has anybody seen it? (Central executive: phonological).* The child questions several witnesses about the color of the absconded thief’s attire. Each of the witnesses states a different color out of nine options possible. Then, a lightbulb appears over the head of the victim who has made the correct statement. The child is now asked to select the correctly stated color on the screen. The harder the task gets the more witnesses state a color.
*Break the Code. (Central executive: visual-spatial).* Initially, a 4-by-4 grid of 20 white squares can be seen. The grid’s four edges appear in different colors (red, yellow, green and blue). In the following, the grid turns 90 degrees counter-clockwise or clockwise and some squares fade to black. This is the code the test person has to decipher. Shortly thereafter, the squares change back to white and the grid rotates to its original position. In order to break the code, the child will have to select the previously black sections by touching the corresponding screen areas. The colored edges of the grid serve as an orientation. The higher the level of difficulty, the more squares fade to black (a maximum of eight).
*Caught Red-handed! (Central executive: visual-spatial).* The child’s initial visual impression is a bird’s eye view of an urban setting with a thief roaming the streets. Gradually, more thieves appear in the streets, indistinguishable in appearance from the first one. On cue, the test person is to mark the thieves in the right order of appearance. A higher level of difficulty increases the number of thieves (a maximum of nine).

The training’s concept as a computer application offers a number of advantages: For one thing, prior to becoming active in the game, the children hear and see the instructions for and an example of the respective game, detailing the strategies for the task ahead. Also, the automatic adjustment of difficulty levels of this training enables every child to continuously work on the individual performance limit (adaptivity). In this vein, the game’s difficulty increases every time the child masters two consecutive trials, the difficulty decreases after three consecutively unsuccessful trials. Moreover, a touchscreen computer as a training device appears to be especially appealing to children, thereby promoting their motivation to participate in the training. The latter has been further encouraged by the implementation of a reward system in the form of a high score, through which the children can earn a certain number of coins depending on their performance.

### 2.4. Strategy of Analyses

For all analyses, the level of significance has been defined as α = 0.05.

To answer the first research question, we examined whether the operational efficiency of working memory can experience a long-term improvement through the performed training. 

For each subtest, 2 (dyslexia vs. control) by 2 (trained vs. untrained) factorial analyses of variance, including repeated measures (pre-test vs. follow-up), were calculated. Working memory performance of the four groups at two test intervals is depicted in Table 2.

The second question was whether predominantly children suffering from dyslexia and an especially poor working memory performance would benefit from training. For this question, means for each subsystem of working memory were defined for all children with dyslexia (not for controls), using the individual working memory tasks of the pre-test. According to the median, the group was divided up into lower vs. higher starting levels. The performances of the respective groups are shown in Table 3.

Based on the approach in question 1, again, a set of 2 (weak vs. strong) × 2 (trained vs. untrained) factorial analyses of variance, including repeated measures (pre-test vs. follow-up), was performed for the subtests.

## 3. Results

For the sake of clarity, all results of the factorial analyses of variance are summarized in Table 4. As to be expected, for the main effect dyslexia (comparison of dyslexic children with control group), significant differences appeared in all tasks of the phonological loop, as well as of the central executive (except for *object span* and *Corsi block backwards*), while differences did not show up in the visuo-spatial working memory tasks. For the observation of a general training effect, the triple effects of interaction (dyslexia, training conditions and time of measure) are of central interest. Neither in the phonological loop tasks, nor in the central executive tasks, could substantial training effects be detected. Solely, the task *Corsi block* exhibits a significant triple interaction. Further analyses clarify that between the two measure intervals, all groups—excepting the untrained group with dyslexia—improve significantly. Hence, improvement cannot be attributed to the training only, since the untrained control groups also made progress.

The second question was whether children suffering from dyslexia and especially poor working memory performance would benefit more from training than dyslexic children with an initially higher performance. As the results in Table 5 illustrate, in all tasks, an expected significant main effect of initial working memory performance becomes evident. 

In contrast, no significant triple interaction between initial working memory performance, training and time of measurement could be detected for the tasks performed. Thus, also in these analyses, no significant training effects could be identified.

## 4. Discussion

The operational efficiency of working memory is an important predictor for future school performance in the fields of reading, writing and mathematics [42]. A current topic of discussion is whether working memory plays a more important role than intelligence in the context of school achievement [43]. Children who have developed learning difficulties in their academic career exhibit specific deficits in working memory functions [11]. In this respect, an intact working memory possesses particular importance as the prerequisite of successful information processing. This insight fosters the notion of positively influencing the operational efficiency of working memory, especially when it comes to children with deficits in this field. Different training programs for working memory improvement have been developed, but as depicted above, the results of several evaluations are quite heterogenous in nature and allow no unequivocal answer to the question whether an efficient and long-lasting improvement of working memory is feasible at all and if so, by which means.

The present study also aimed at developing and evaluating a training program. Relying on ten different computer-assisted games, designed for the phonological loop, the visuo-spatial sketchpad and the central executive, the working memory performance of elementary school children was to be improved. While children unaffected by dyslexia participated in the study, the emphasis was on children with dyslexia. The results once more exemplify the opportunities, but even more so the limits of such an undertaking: Neither dyslexia-unaffected nor -affected children have been able to experience long-term improvement in working memory performance—only the visuo-spatial Corsi block yielded substantial training results. Also, for children with dyslexia and especially disadvantageous initial working memory performance regarding the three subsystems of working memory, no performance increase through training could be determined. Hence, very intensive practice (tri-weekly sessions of 45 minutes over a course of six weeks) does not enable the children to process and memorize greater quantities of information over a longer period [37].

Especially intriguing is the fact that the segment of the phonological loop did not exhibit any training effect at all—neither with dyslexia-unaffected nor with -affected children. This was particularly disappointing, because the training featured a greater portion of games designed for the improvement of the phonological loop. The intention behind this focus was to equip dyslexic children with the tools necessary—in the form of extensive training opportunities—to tackle their causal primary working memory deficit. The fact that this did not even result in temporary increases, or in long term maintenance, suggests that the memorization and processing of sounds and acoustical information must, in all probability, be neurocognitively ingrained especially deep. If at all, improvement is only conceivable through a much higher intensity of training input. (This idea is supported by the remarkably well-developed phonological working memory abilities of blind persons. Due to the unavailability of visual input, these individuals have to process a far greater amount of acoustical information; [44].) For children with dyslexia, this is a disappointing result—especially for this group, progress in this field would have been of crucial importance. Furthermore, the following problems have to remain unsolved: On the one hand, we cannot be sure whether any training success might have any positive effect on transfer or other cognitive performance (which are less closely associated with the training). On the other hand: Does a better working memory performance really result in improved academic performance and ideally contribute to the solution of written language problems? We cannot answer that question because we did not find a positive training effect that might transfer to school achievement. Nevertheless, in this study, we collected data on possible increase of school achievement (reading, writing and math) but again we did not find any effects of the training. (Given the lack of a training effect of AGENT 8–1–0 on working memory performance at post-test and follow-up, the question of a transfer on school achievement did not really make sense and, therefore, the analyses are not reported in detail.) The transfer problem also became obvious in a meta-analysis by Melby-Lervåg and Hulme [34]: Repeatedly, only short-term transfer effects and no distal transfer effects regarding content could be produced for school performance.

Within the present controversy over the trainability of working memory, the results seem to support the sceptics regarding the efficacy of such endeavors. Of course, it is imaginable that the training did not succeed as desired because it failed to properly motivate the children for working at their individual limits of performance. There is also the possibility that the number of training sessions—the period of six weeks, or training time per week—was not sufficient to effect changes. However, the games were closely oriented towards the theoretical construct defined by Baddeley [8], and many different aspects of working memory activity have been precisely reflected in the games, but with meager results. The few short-term effects discernible at the end of the training [37] were lost after a couple of months. The fact that there have been no training effects whatsoever in the phonological loop is a particularly disappointing one. This would have been of special importance for children with dyslexia, since their specific working memory deficit can be localized within the phonological loop.

To conclude, while keeping in mind this study’s hardly optimistic results, the question about practical implications arises. If, according to these results, a long-term or even permanent improvement of the operational efficiency of working memory is not feasible, it does not constitute a noteworthy problem for children with an intact working memory. However, for children suffering from dyslexia, who in this study and in many others, exhibit poor working memory functions, the need for compensatory strategies arises. If increases in capacity via memory expansion or an improved automatic processing appear to be impossible, an intervention must focus on the remaining determinants of memory capacity, especially on memory strategies and meta-memory [29]. For children with dyslexia, the recommendation of a functional exercise treatment for reading and writing remains valid; this should specifically include applied strategies and self-monitoring of the individual reading and writing process.

## Figures and Tables

**Table 1:** Sex distribution and means (standard deviations) of age, IQ, spelling t-scores and reading t-scores by subgroup.

	C-T (n = 27)	C-UT (n = 28)	DYS-T (n = 43)	DYS-UT (n = 41)
Sex (m/w)	15/12	16/12	26/17	29/12
Age (years)	8.82 (0.43)	8.55 (0.34)	9.08 (0.75)	8.77 (0.49)
IQ	107.26 (14.40)	101.93 (6.93)	98.50 (12.91)	100.73 (13.05)
Reading—*T*-Wert	51.65 (8.32)	51.55 (3.62)	40.42 (8.33)	41.40 (8.10)
Spelling—*T*-Wert	48.96 (8.36)	50.79 (4.86)	37.00 (6.54)	36.90 (4.31)

Note: C-T = control group trained; C-UT = control group untrained; DYS-T = dyslexia trained; DYS-UT = dyslexia untrained.

**Table 2:** Means (standard deviations) for working memory measures by subgroup.

	C-T		C-UT		DYS-T		DYS-UT	
Working Memory	Pre-Test	Follow-Up	Pre-Test	Follow-Up	Pre-Test	Follow-Up	Pre-Test	Follow-Up
**Phonological Loop**								
Digit Span	4.89 (0.66)	5.04 (0.82)	4.88 (0.54)	5.36 (0.76)	4.37 (0.58)	4.74 (0.61)	4.42 (0.60)	4.83 (0.69)
One-Syllable Word Span	4.19 (0.70)	4.67 (0.78)	4.43 (0.62)	4.66 (0.69)	3.86 (0.54)	4.38 (0.69)	4.05 (0.60)	4.34 (0.79)
Three-Syllable Word Span	3.50 (0.57)	3.78 (0.74)	3.36 (0.45)	3.54 (0.59)	3.23 (0.45)	3.30 (0.50)	3.20 (0.45)	3.35 (0.61)
**Visual-Spatial Sketchpad**								
Corsi Block Span	4.85 (0.74)	5.26 (0.63)	4.61 (0.64)	5.32 (0.64)	4.58 (0.58)	5.13 (0.84)	4.82 (0.64)	4.98 (0.62)
Matrix Span	5.57 (1.44)	6.57 (1.28)	5.29 (1.14)	6.70 (1.26)	5.30 (1.28)	6.39 (1.42)	5.39 (1.31)	6.16 (1.24)
**Central Executive**								
Backward Digit Span	3.72 (0.53)	4.13 (0.86)	3.82 (0.76)	4.11 (0.72)	3.42 (0.58)	3.68 (0.66)	3.38 (0.47)	3.55 (0.71)
Backward Words Span	3.41 (0.48)	3.70 (0.84)	3.50 (0.61)	3.77 (0.99)	2.95 (0.76)	3.22 (0.62)	3.18 (0.48)	3.22 (0.72)
Color Span Backwards	3.35 (0.89)	3.91 (0.89)	3.66 (0.97)	4.14 (0.78)	2.88 (0.71)	3.55 (0.97)	3.35 (0.71)	3.65 (0.83)
Corsi Block Span Backwards	4.41 (0.84)	4.80 (0.84)	4.38 (0.73)	4.89 (0.42)	4.13 (0.84)	4.57 (0.95)	4.34 (0.68)	4.82 (0.58)
Object Span	3.20 (0.94)	3.91 (0.87)	3.55 (0.75)	4.43 (0.77)	3.36 (0.83)	3.61 (0.94)	3.54 (0.73)	3.50 (0.91)
Counting Span	3.83 (0.92)	4.24 (1.01)	3.89 (0.79)	4.24 (1.01)	3.14 (0.82)	3.28 (0.73)	3.49 (0.93)	3.71 (0.89)

*Note.* C-T = control group trained; C-UT = control group untrained; DYS-T = dyslexia trained; DYS-UT = dyslexia untrained.

**Table 3:** Means (standard deviations) for working memory measures by subgroup.

	DYS-Weak-T		DYS-Weak-UT		DYS-Strong-T		DYS-Strong-UT	
Working Memory	Pre-Test	Follow-Up	Pre-Test	Follow-Up	Pre-Test	Follow-Up	Pre-Test	Follow-Up
**Phonological Loop**								
Digit Span	4.00 (0.43)	4.47 (0.57)	3.93 (0.34)	4.50 (0.63)	4.70 (0.49)	4.95 (0.57)	4.88 (0.38)	5.14 (0.60)
One-Syllable Word Span	3.48 (0.44)	4.03 (0.65)	3.65 (0.46)	3.95 (0.65)	4.20 (0.36)	4.67 (0.60)	4.43 (0.46)	4.71 (0.73)
Three-Syllable Word Span	2.93 (0.18)	3.09 (0.20)	2.93 (0.18)	3.10 (0.26)	3.50 (0.45)	3.48 (0.60)	3.45 (0.47)	3.60 (0.74)
**Visual-Spatial Sketchpad**								
Corsi Block Span	4.38 (0.54)	4.91 (0.77)	4.48 (0.56)	4.79 (0.54)	4.84 (0.53)	5.41 (0.87)	5.18 (0.52)	5.18 (0.65)
Matrix Span	4.44 (0.88)	6.02 (1.46)	4.43 (0.75)	5.83 (1.12)	6.40 (0.76)	6.85 (1.27)	6.40 (0.97)	6.50 (1.29)

**Table 4:** Results (ANOVA).

Working Memory	Main Effect Dyslexia	Main Effect Training	Main Effect Time of Measurement	Interaction Time of Measurement × Dyslexia	Interaction Time of Measurement × Training	Interaction Dyslexia × Training	Interaction Time of Measurement × Dyslexia × Training
**Phonological Loop**							
Digit Span	F(1;130) = 20.74	F(1;130)=1.31	F(1;130) = 43.45	F(1;130) = 2.88	F(1;130) < 1	F(1;130) < 1	F(1;130) = 1.92
	*p* < 0.001	*p* = 0.254	*p* < 0.001	*p* = 0.092	*p* = 0.929	*p* = 0.700	*p* = 0.167
	*ηp*^2^ = 0.138	*ηp*^2^ = 0.010	*ηp*^2^ = 0.250	*ηp*^2^ = 0.022	*ηp*^2^ = 0.000	*ηp*^2^ = 0.001	*ηp*^2^ = 0.015
One-Syllable Word Span	F(1;130) = 9.58	F(1;130) < 1	F(1;130) = 38.99	F(1;130) < 1	F(1;130) = 3.44	F(1;130) < 1	F(1;130) < 1
	*p* = 0.002	*p* = 0.395	*p* < 0.001	*p* = 0.783	*p* = 0.066	*p* = 0.765	*p* = 0.818
	*ηp*^2^ = 0.069	*ηp*^2^ = 0.006	*ηp*^2^ = 0.231	*ηp*^2^ = 0.001	*ηp*^2^ = 0.026	*ηp*^2^ = 0.001	*ηp*^2^ = 0.000
Three-Syllable Word Span	F(1;130) = 10.46	F(1;130) = 1.66	F(1;130) = 9.63	F(1;130) = 1.73	F(1;130) < 1	F(1;130) = 1.21	F(1;130) = 1.25
	*p* = 0.002	*p* = 0.200	*p* = 0.193	*p* = 0.191	*p* = 0.073	*p* = 0.273	*p* = 0.265
	*ηp*^2^ = 0.031	*ηp*^2^ = 0.013	*ηp*^2^ = 0.013	*ηp*^2^ = 0.013	*ηp*^2^ = 0.000	*ηp*^2^ = 0.009	*ηp*^2^ = 0.010
**Visual Spatial Sketchpad**							
Corsi Block Span	F(1;130) = 1.63	F(1;130) < 1	F(1;130) = 40.47	F(1;130) = 2.47	F(1;130) < 1	F(1;130) < 1	F(1;130) = 5.37
	*p* = 0.204	*p* = 0.687	*p* < 0.001	*p* = 0.10	*p* = 0.90	*p* = 0.557	*p* = 0.022
	*ηp*^2^ = 0.012	*ηp*^2^ = 0.001	*ηp*^2^ = 0.237	*ηp*^2^ = 0.021	*ηp*^2^ = 0.000	*ηp*^2^ = 0.002	*ηp*^2^ = 0.040
Matrix Span	F(1;130) = 1.35	F(1;130) < 1	F(1;130) = 63.74	F(1;130) = 1.78	F(1;130) < 1	F(1;130) < 1	F(1;130) = 1.78
	*p* = 0.247	*p* = 0.645	*p* = 0.001	*p* = 0.280	*p* = 0.832	*p* = 0.990	*p* = 0.185
	*ηp*^2^ =0.010	*ηp*^2^ = 0.002	*ηp*^2^ = 0.329	*ηp*^2^ = 0.009	*ηp*^2^ = 0.000	*ηp*^2^ = 0.000	*ηp*^2^ = 0.013
**Central Executive**							
Digit Span Backwards	F(1;130) = 21.70	F(1;130) < 1	F(1;130) = 14.94	F(1;130) = 1.01	F(1;130) < 1	F(1;130) < 1	F(1;130) < 1
	*p* = 0.001	*p* = 0.730	*p* = 0.000	*p* = 0.318	*p* = 0.551	*p* = 0.448	*p* = 0.846
	*ηp*^2^ = 0.143	*ηp*^2^ = 0.001	*ηp*^2^ = 0.103	*ηp*^2^ = 0.004	*ηp*^2^ = 0.003	*ηp*^2^ = 0.004	*ηp*^2^ = 0.000
Word Span Backwards	F(1;130) = 21.61	F(1;130) = 1.11	F(1;130) = 9.41	F(1;130) < 1	F(1;130) = 1.00	F(1;130) < 1	F(1;130) < 1
	*p* = 0.001	*p* = 0.295	*p* = 0.003	*p* = 0.446	*p* = 0.319	*p* = 0.798	*p* = 0.421
	*ηp*^2^ = 0.143	*ηp*^2^ = 0.008	*ηp*^2^ = 0.067	*ηp*^2^ = 0.004	*ηp*^2^ = 0.008	*ηp*^2^ = 0.001	*ηp*^2^ = 0.005
Color Span Backwards	F(1;130) = 10.87	F(1;130) = 5.13	F(1;130) = 40.59	F(1;130) < 1	F(1;130) = 2.26	F(1;130) < 1	F(1;130) = 1.08
	*p* = 0.001	*p* = 0.025	*p* = 0.000	*p* = 0.881	*p* = 0.135	*p* = 0.924	*p* = 0.300
	*ηp*^2^ = 0.077	*ηp*^2^ = 0.038	*ηp*^2^ = 0.238	*ηp*^2^ = 0.000	*ηp*^2^ = 0.017	*ηp*^2^ = 0.000	*ηp*^2^ = 0.008
Corsi Block Backwards	F(1;130) = 1.83	F(1;130) < 1	F(1;130) = 48.98	F(1;130) < 1	F(1;130) < 1	F(1;130) < 1	F(1;130) < 1
	*p* = 0.178	*p* = 0.328	*p* = 0.000	*p* = 0.997	*p* = 0.750	*p* = 0.435	*p* = 0.770
	*ηp*^2^ = 0.014	*ηp*^2^ = 0.007	*ηp*^2^ = 0.278	*ηp*^2^ = 0.000	*ηp*^2^ = 0.001	*ηp*^2^ = 0.005	*ηp*^2^ = 0.001
Object Span	F(1;130) = 1.08	F(1;130) < 1	F(1;130) = 10.02	F(1;130) = 3.80	F(1;130) = 4.08	F(1;130) < 1	F(1;130) < 1
	*p* = 0.301	*p* = 0.474	*p* = 0.002	*p* = 0.053	*p* = 0.045	*p* = 0.737	*p* = 0.680
	*ηp*^2^ = 0.008	*ηp*^2^ = 0.004	*ηp*^2^ = 0.072	*ηp*^2^ = 0.028	*ηp*^2^ = 0.030	*ηp*^2^ = 0.001	*ηp*^2^ = 0.001
Counting Span	F(1;130) = 29.02	F(1;130) = 3.60	F(1;130) = 13.57	F(1;130) = 3.89	F(1;130) < 1	F(1;130) < 1	F(1;130) < 1
	*p* = 0.001	*p* = 0.060	*p* = 0.000	*p* = 0.051	*p* = 0.399	*p* = 0.362	*p* = 0.939
	*ηp*^2^ = 0.183	*ηp*^2^ = 0.027	*ηp*^2^ = 0.095	*ηp*^2^ = 0.029	*ηp*^2^ = 0.000	*ηp*^2^ = 0.006	*ηp*^2^ = 0.000

**Table 5:** Results (ANOVA) median split.

Working Memory	Main Effect Dyslexia	Main Effect Training	Main Effect Time of Measurement	Interaction Time of Measurement × Dyslexia	Interaction Time of Measurement × Training	Interaction Dyslexia × Training	Interaction Time of Measurement × Dyslexia × Training
**Phonological Loop**							
Digit Span	F(1;75) = 53.54	F(1;75) = 1.06	F(1;75) = 40.18	F(1;75) = 3.45	F(1;75) < 1	F(1;75) = 1.62	F(1;75) < 1
	*p* < 0.001	*p* = 0.307	*p* < 0.001	*p* = 0.067	*p* = 0.824	*p* = 0.207	*p* = 0.553
	*ηp*^2^ = 0.417	*ηp*^2^ = 0.014	*ηp*^2^ = 0.349	*ηp*^2^ = 0.044	*ηp*^2^ = 0.001	*ηp*^2^ = 0.021	*ηp*^2^ = 0.005
One-Syllable Word Span	F(1;75) = 50.43	F(1;75) < 1	F(1;75) = 28.07	F(1;75) < 1	F(1;75) = 1.79	F(1;75) < 1	F(1;75) < 1
	*p* < 0.001	*p* = 0.417	*p* < 0.001	*p* = 0.758	*p* = 0.185	*p* = 0.640	*p* = 0.833
	*ηp*^2^ = 0.402	*ηp*^2^ = 0.009	*ηp*^2^ = 0.272	*ηp*^2^ = 0.001	*ηp*^2^ = 0.023	*ηp*^2^ = 0.003	*ηp*^2^ = 0.001
Three-Syllable Word Span	F(1;75) = 43.77	F(1;75) < 1	F(1;75) = 2.30	F(1;75) < 1	F(1;75) < 1	F(1;75) < 1	F(1;75) < 1
	*p* < 0.001	*p* = 0.949	*p* = 0.134	*p* = 0.337	*p* = 0.353	*p* = 0.926	*p* = 0.862
	*ηp*^2^ = 0.369	*ηp*^2^ = 0.000	*ηp*^2^ = 0.030	*ηp*^2^ = 0.012	*ηp*^2^ = 0.011	*ηp*^2^ = 0.000	*ηp*^2^ = 0.000
**Visual Spatial Sketchpad**							
Corsi Block Span	F(1;75) = 24.28	F(1;75) < 1	F(1;75) = 11.62	F(1;75) < 1	F(1;75) = 3.26	F(1;75) < 1	F(1;75) < 1
	*p* < 0.001	*p* = 0.970	*p* < 0.001	*p* = 0.509	*p* = 0.075	*p* = 0.760	*p* = 0.350
	*ηp*^2^ = 0.245	*ηp*^2^ = 0.000	*ηp*^2^ = 0.134	*ηp*^2^ = 0.006	*ηp*^2^ = 0.042	*ηp*^2^ = 0.001	*ηp*^2^ = 0.012
Matrix Span	F(1;75) = 46.67	F(1;75) < 1	F(1;75) = 32.48	F(1;75) = 13.46	F(1;75) < 1	F(1;75) < 1	F(1;75) < 1
	*p* < 0.001	*p* = 0.468	*p* = 0.001	*p* < 0.001	*p* = 0.400	*p* = 0.990	*p* = 0.592
	*ηp*^2^ =0.384	*ηp*^2^ = 0.007	*ηp*^2^ = 0.302	*ηp*^2^ = 0.152	*ηp*^2^ = 0.009	*ηp*^2^ = 0.000	*ηp*^2^ = 0.004
**Central Executive**							
Digit Span Backwards	F(1;75) = 12.10	F(1;75) = 3.49	F(1;75) = 4.70	F(1;75) = 1.10	F(1;75) < 1	F(1;75) = 1.71	F(1;75) < 1
	*p* = 0.008	*p* = 0.646	*p* = 0.079	*p* = 0.273	*p* = 0.283	*p* = 0.070	*p* = 0.690
	*ηp*^2^ = 0.139	*ηp*^2^ = 0.044	*ηp*^2^ = 0.059	*ηp*^2^ = 0.014	*ηp*^2^ = 0.000	*ηp*^2^ = 0.022	*ηp*^2^ = 0.000
Word Span Backwards	F(1;75) = 7.48	F(1;75) < 1	F(1;75) = 3.17	F(1;75) = 1.22	F(1;75) = 1.17	F(1;75) = 3.38	F(1;75) < 1
	*p* = 0.001	*p* = 0.295	*p* = 0.003	*p* = 0.446	*p* = 0.319	*p* = 0.798	*p* = 0.421
	*ηp*^2^ = 0.091	*ηp*^2^ = 0.003	*ηp*^2^ = 0.041	*ηp*^2^ = 0.016	*ηp*^2^ = 0.015	*ηp*^2^ = 0.043	*ηp*^2^ = 0.002
Color Span Backwards	F(1;75) = 13.31	F(1;75) < 1	F(1;75) = 355.36	F(1;75) < 1	F(1;75) = 5.14	F(1;75) = 1.40	F(1;75) < 1
	*p* < 0.001	*p* = 0.927	*p* < 0.001	*p* = 0.470	*p* = 0.026	*p* = 0.241	*p* = 0.392
	*ηp*^2^ = 0.151	*ηp*^2^ = 0.000	*ηp*^2^ = 0.335	*ηp*^2^ = 0.001	*ηp*^2^ = 0.064	*ηp*^2^ = 0.018	*ηp*^2^ = 0.010
Corsi Block Backwards	F(1;75) = 26.65	F(1;75) < 1	F(1;75) = 15.16	F(1;75) = 3.06	F(1;75) < 1	F(1;75) = 2.58	F(1;75) < 1
	*p* < 0.001	*p* = 0.553	*p* < 0.001	*p* = 0.084	*p* = 0.482	*p* = 0.112	*p* = 0.674
	*ηp*^2^ = 0.197	*ηp*^2^ = 0.005	*ηp*^2^ = 0.168	*ηp*^2^ = 0.039	*ηp*^2^ = 0.007	*ηp*^2^ = 0.033	*ηp*^2^ = 0.002
Object Span	F(1;75) = 18.34	F(1;75) < 1	F(1;75) = 1.15	F(1;75) = 1.07	F(1;75) < 1	F(1;75) = 2.63	F(1;75) < 1
	*p* < 0.001	*p* = 0.460	*p* = 0.287	*p* = 0.304	*p* = 0.363	*p* = 0.109	*p* = 0.342
	*ηp*^2^ = 0.197	*ηp*^2^ = 0.007	*ηp*^2^ = 0.015	*ηp*^2^ = 0.014	*ηp*^2^ = 0.011	*ηp*^2^ = 0.034	*ηp*^2^ = 0.012
Counting Span	F(1;75) = 21.60	F(1;75) = 1.84	F(1;75) = 1.84	F(1;75) = 14.71	F(1;75) = 2.66	F(1;75) < 1	F(1;75) < 1
	*p* < 0.001	*p* = 0.179	*p* = 0.180	*p* < 0.001	*p* = 0.107	*p* = 0.421	*p* = 0.651
	*ηp*^2^ = 0.224	*ηp*^2^ = 0.024	*ηp*^2^ = 0.024	*ηp*^2^ = 0.164	*ηp*^2^ = 0.034	*ηp*^2^ = 0.009	*ηp*^2^ = 0.003

## References

[1] Fletcher J.M., Lyon G.R., Fuchs L.S., Barnes M.A. (2018). Learning Disabilities: From Identification to Intervention.

[2] Weber J., Marx P., Schneider W., Hasselhorn M. (2008). Lese-Rechtschreibschwierigkeiten. Handbuch der Pädagogischen Psychologie.

[3] Hasselhorn M., Schuchardt K. (2006). Lernstörungen: Eine kritische skizze zur epidemiologie. Kindh. Entwickl..

[4] Pickering S.J. (2006). Working Memory and Education.

[5] Swanson H.L., Alloway T.P., Gathercole S.E. (2006). Working Memory and Reading Disabilities: Both Phonological and Executive Processing Deficits are Important. Working Memory and Neurodevelopmental Disorders.

[6] Vellutino F.R., Fletcher J.M., Snowling M.J., Scanlon D.M. (2004). Specific reading disability (dyslexia): What have we learned in the past four decades. J. Child Psychol. Psychiatry.

[7] Wagner R.K., Torgesen J.K. (1987). The nature of phonological processing and ist causal role in the acquisition of reading skills. Psychol. Bull..

[8] Baddeley A.D. (1986). Working Memory.

[9] Baddeley A.D., Hitch G.J., Bower G.H. (1974). Working Memory. The Psychology of Learning and Motivation: Advances in Research and Theory.

[10] Alloway T.P., Gathercole S.E. (2006). Working Memory and Neurodevelopmental Disorders.

[11] Schuchardt K., Mähler C., Hasselhorn M. (2008). Working memory deficits in children with different learning disabilities. J. Learn. Disabil..

[12] Baddeley A.D., Pickering S.J. (2006). Working Memory: An Overview. Working Memory and Education.

[13] Baddeley A.D. (1996). Exploring the central executive. Q. J. Exp. Psychol..

[14] Case R.D., Kurland M., Goldberg J. (1982). Operational efficiency and the growth of short-term memory span. J. Exp. Child Psychol..

[15] Daneman M., Carpenter P.A. (1980). Individual differences in working memory and reading. J. Verbal Learn. Verbal Behav..

[16] Gathercole S.E., Pickering S.J. (2001). Working memory deficits in children with special educational needs. Br. J. Spec. Educ..

[17] Brandenburg J., Klesczewski J., Fischbach A., Schuchardt K., Büttner G., Hasselhorn M. (2015). Working memory in children with learning disabilities in reading versus spelling: Searching for overlapping and specific cognitive factors. J. Learn. Disabil..

[18] Hasselhorn M., Schuchardt K., Mähler C. (2010). Phonologisches Arbeitsgedächtnis bei Kindern mit diagnostizierter Lese- und/oder Rechtschreibstörung: Zum Einfluss von Wortlänge und Lexikalität auf die Gedächtnisspanne. Z. Entwicklungspsychol. Pädagog. Psychol..

[19] Landerl K., Bevan A., Butterworth B. (2004). Developmental dyscalculia and basic numerical capacities: A study of 8-9-year-old students. Cognition.

[20] Palmer S. (2000). Phonological recoding deficit in working memory of dyslexic teenagers. J. Res. Read..

[21] Pickering S.J., Gathercole S.E., Gathercole S., Alloway T. (2005). Working Memory Deficits in Dyslexia: Are They Located in the Phonological Loop, Visuospatial Sketchpad or Central Executive?. Working Memory and Neurodevelopmental Disorders.

[22] Siegel L.S., Ryan E.B. (1989). The development of working memory in normally achieving and subtypes of learning-disabled children. Child Dev..

[23] Swanson H.L. (1993). Working memory in learning disability subgroups. J. Exp. Child Psychol..

[24] Swanson H.L. (1999). Reading comprehension and working memory in learning-disabled readers: Is the phonological loop more important than the executive system?. J. Exp. Child Psychol..

[25] Eden G.F., Stein J.F. (1995). Verbal and visual problems in reading disability. J. Learn. Disabil..

[26] Howes N.L., Bigler E.D., Burlingame G.M., Lawson J.S. (2003). Memory performance of children with dyslexia. J. Learn. Disabil..

[27] Kibby M., Marks W., Morgan S., Long C. (2004). Specific impairment in developmental reading disabilities: A working memory approach. J. Learn. Disabil..

[28] O’Shaughnessy T., Swanson H.L. (1998). Do immediate memory deficits in students with learning disabilities in reading reflect a developmental lag or deficit? A selective meta-analysis of the literature. Learn. Disabil. Q..

[29] Mähler C., Hasselhorn M., Klauer K.J. (2001). Lern- und Gedächtnistraining bei Kindern. Handbuch Kognitives Training.

[30] Klingberg T., Fernell E., Olesen P., Johnson M., Gustafsson P., Dahlström K., Gillberg C.G., Forssberg H., Westerberg H. (2005). Computerized training of working memory in children with ADHD—A controlled, randomized, double-blind trial. J. Am. Acad. Child Adolesc. Psychiatry.

[31] Sala G., Gobet F. (2017). Working memory training in typically developing children: A meta-analysis of the available evidence. Dev. Psychol..

[32] Holmes J., Gathercole S.E., Dunning D.L. (2009). Adaptive training leads to sustained enhancemant of poor working memory in children. Dev. Sci..

[33] Thorell L.B., Lindquist S., Bergmann Nutley S., Bohlin G., Klingberg T. (2009). Training and transfer effects of executive functions in preschool children. Dev. Sci..

[34] Melby-Lervåg M., Hulme C. (2012). Is working memory training effective? A meta-analytic review. Dev. Psychol..

[35] Alloway T.P. (2012). Can interactive working memory training improve learning?. JILR.

[36] Shipstead Z., Hicks K.L., Engle R.W. (2012). Working memory training remains a work in progress. J. Appl. Res. Mem. Cogn..

[37] Mähler C., Jörns C., Radtke E., Schuchardt K. (2015). Chancen und Grenzen eines Trainings des Arbeitsgedächtnisses bei Kindern mit und ohne Lese-/Rechtschreibschwierigkeiten. Z. Erziehwiss..

[38] Cattell R., Weiß R.H., Osterland J. (1997). Culture Fair Test—Grundintelligenztest Skala 1 (CFT 1).

[39] Lenhardt W., Schneider W. (2006). Ein Leseverständnistest für Erst- bis Sechstklässler (ELFE 1–6).

[40] Birkel P. (2007). Weingartener Grundwortschatz Rechtschreib-Test für 2. und 3. Klassen (WRT 2+).

[41] Hasselhorn M., Schumann-Hengsteler R., Grube D., König J., Mähler C., Schmid I., Seitz-Stein K., Zoelch C. (2012). Arbeitsgedächtnistestbatterie für Kinder von 5 bis 12 Jahren (AGTB 5-12).

[42] Mähler C., Piekny J., Goldammer A., Balke-Melcher C., Schuchardt K., Grube D., Cloos P., Koch K., Mähler C. (2015). Kognitive Kompetenzen als Prädiktoren für Schulleistungen im Grundschulalter. Entwicklung und Förderung in der frühen Kindheit. Interdisziplinäre Perspektiven.

[43] Alloway T.P., Alloway R.G. (2010). Investigating the predictive roles of working memory and IQ in academic attainment. J. Exp. Child Psychol..

[44] Einecke B., Woerner W., Hasselhorn M., Hasselhorn M., Zoelch C. (2012). Funktionstüchtigkeit des Phonologischen Arbeitsgedächtnisses bei blinden Kindern im Grundschulalter. Funktionsdiagnostik des Arbeitsgedächtnisses. Tests und Trends.

